# Identification of the O-Glycan Epitope Targeted by the Anti-Human Carcinoma Monoclonal Antibody (mAb) NEO-201

**DOI:** 10.3390/cancers14204999

**Published:** 2022-10-12

**Authors:** Kwong Y. Tsang, Massimo Fantini, Anjum Zaki, Sharon A. Mavroukakis, Maria Pia Morelli, Christina M. Annunziata, Philip M. Arlen

**Affiliations:** 1 Precision Biologics, Inc., Bethesda, MD 20814, USA; 2 Women’s Malignancy Branch, Center for Cancer Research, National Cancer Institute, National Institutes of Health, Bethesda, MD 20892, USA

**Keywords:** O-glycan, monoclonal antibody, antibody-dependent cellular cytotoxicity, NEO-201, cancer immunotherapy

## Abstract

**Simple Summary:**

Glycosylation is an important post-translational modification made on mammalian proteins and lipids. In cancer cells, the disruption of several glycosylation patterns, such as the O-glycosylation, has been observed. The expression of incomplete/truncated O-glycans in cancer cells occurs in both solid and liquid tumors and is correlated with poor prognosis and tumor progression. The employment of monoclonal antibodies (mAbs) targeting truncated O-glycans in cancer cells could serve as an effective strategy to counteract tumor growth. In previous studies, we reported that the IgG1-humanized mAb NEO-201 binds specifically to tumor-associated variants of CEACAM5 and CEACAM6 expressed by colon, ovarian, pancreatic, non-small cell lung, head and neck, cervical, uterine and breast cancers but is not reactive against most normal tissues. Since CEACAMs are highly glycosylated proteins, in this article, we evaluated whether the epitope recognized by NEO-201 is an O-glycan. This study demonstrated that NEO-201 binds to core 1 O-glycans and targets and kills cancer cells expressing core 1 and extended core 1 O-glycans. Usually, GalNAc residue can be added on to threonine and serine to form O-glycans, suggesting that NEO-201 binds to core 1 and extended core 1 O-glycans attached to any protein carrying amino acid regions containing serine and threonine

**Abstract:**

Truncated O-glycans expressed in cancer cells support tumor progression, and they may serve as potential targets to improve the monitoring and treatment of cancers. Previously, we reported that NEO-201 binds to several tumors expressing tumor-associated CEACAM5 and CEACAM6 variants but does not bind to those expressed in healthy tissues. This specific binding may be associated with the presence of truncated O-glycans attached on the protein sequence of these variants. To evaluate the glycosylation pattern targeted by NEO-201 we performed an O-glycan array consisting of 94 O-glycans. O-glycan profiles were elucidated from the human pancreatic cancer cell line CFPAC-1, human hematological neoplastic cells (HL-60, U937, K562) and human neutrophils. The O-glycan array analysis showed that NEO-201 interacts with core 1-4 O-glycans and that the binding to a specific core 1 O-glycan was the strongest. The O-glycan profiling of the NEO-201-reactive cells CFPAC-1, HL-60, U937 and human neutrophils showed that cells recognized by NEO-201 express mostly core 1 and/or extended core 1 O-glycans. In addition, NEO-201 mediates antibody-dependent cell-mediated cytotoxicity (ADCC) against tumor cells expressing core 1 or extended core 1 O-glycan profiles. These results demonstrated that NEO-201 binds to core 1 and extended core 1 O-glycans expressed in its target cells. Since GalNAc residue can be added onto threonine and serine to form O-glycans, it is very likely that NEO-201 recognizes these O-glycans attached to any protein with amino acid regions containing serine and threonine.

## 1. Introduction

In previous studies, we reported that the humanized IgG1 mAb NEO-201 reacts against numerous carcinomas expressing tumor-associated variants of CEACAM5 and CEACAM6, but it is not reactive against most normal epithelial tissues. NEO-201 can mediate antitumor activity through different mechanisms such as antibody-dependent cellular cytotoxicity (ADCC), complement-dependent cytotoxicity (CDC), and the blockade of the CEACAM5/CEACAM1 immune checkpoint inhibitory pathway [1,2,3,4]. 

Flow cytometry analysis has demonstrated that the target recognized by NEO-201 is expressed in CD15^+^ granulocytes [4], human regulatory T (Treg) cells [5] and several human hematopoietic neoplastic cell lines, including acute myeloid leukemia (AML) cell lines (HL-60, U937) and multiple myeloma (MM) cell lines (OPM2, MM1.S) [6]. NEO-201 does not bind to other immune subsets, such as NK cells, monocytes, B cells, CD8^+^ T cells and a majority of CD4^+^ T cells [4]. Previous studies also showed that NEO-201 attenuates the growth of human tumor xenografts in mice and demonstrates safety/tolerability in non-human primates, with a transient decrease in neutrophils being the only adverse effect observed [1,2].

CEACAMs proteins, such as CEACAM5 and CEACAM6, are highly glycosylated proteins involved in cell migration, metastasis and drug resistance [7]. The specific binding of NEO-201 to tumor-associated variants of CEACAM5 and CEACAM6 could be due to post-translational modifications (i.e., attachment of O-glycans to the protein structure of those proteins) occurring during the process of carcinogenesis. This hypothesis was confirmed in this study, since we observed that NEO-201 binds to mammalian-expressed recombinant human (rh) CEACAM6 (carrying post-translational modifications) but not to bacterial-expressed rhCEACAM6 (devoid of post-translational modifications).

Glycosylation is an important post-translational modification of proteins and lipids and is affected by oncogenesis. In normal cells, at the protein level, glycans can be attached to up to 20 types of amino acids, and the two main glycosylation patterns include amide linkages to asparagine residues (N-glycosylation) and glycosidic linkages to serine and threonine (Ser/Thr) side chains (O-glycosylation) [8]. This O-glycosylation is frequently branched and is very often capped by sialic acid [9].

The O-glycosylation pathway starts with the addition of a single N-acetyl galactosamine (GalNAc) to serine or threonine residue, thus forming the Tn antigen epitope [8]. 

The Tn antigen can be further elongated with galactose to form the T antigen, also referred to as core 1 (Thomsen–Friedenreich antigen). The Tn antigen can also be elongated with N-acetylglucosamine (GlcNAc) to form core 3. The core 1 O-glycans in healthy cells are generally further extended with GlcNAc to form complex branched core 2. Tn can also be elongated with two GlcNAcs to form the core 4 structure. The core 3 and core 4 structures are exclusively present in the intestinal tract [10,11]. In cancer cells, several glycan changes may occur, including the expression of incomplete/truncated glycan structures or increased levels of precursor structures [8,12]. 

Human cancer cells express truncated O-glycans such as Tn antigen and T antigen on the cell surface. These truncated structures can be sialyated to form sialyl-Tn antigen (sTn) and sialyl- or disialyl-T (sT) antigen, which prevents further elongation of the O-glycan structure [8]. The core 1–core 4 O-glycans have been shown to occur as extended complex O-glycans [13]. The Tn and T antigens and sTn and sT antigens are expressed by multiple tumor types, especially those of epithelial origin, such as breast, ovarian, gastric, pancreatic and colon cancers [14,15,16]. Truncated O-glycans, in general, support tumor progression, and their presence is strongly correlated with a poor prognosis [17,18]. 

To evaluate if NEO-201 recognizes O-glycans on cancer cells, we performed an O-glycan array with 94 O-glycans, and then we evaluated if O-glycans recognized by NEO-201 were expressed in cancer cell lines positive for NEO-201 binding by flow cytometry. This study demonstrated that NEO-201 strongly binds to core 1 and/or extended core 1 O-glycans that can be attached to protein carriers on NEO-201 target cells. 

## 2. Materials and Methods

### 2.1. Peripheral Blood Mononuclear Cells (PBMCs)

PBMCs from healthy volunteer donors were obtained from the National Institutes of Health (NIH) Clinical Center Blood Bank (NCT00001846) under the appropriate Institutional Review Board approval and informed consent. 

### 2.2. Reagents 

The NEO-201 mAb employed in this study was generated from the Hollinshead vaccine platform, using tumor-associated antigens derived from tumor membrane fractions pooled from surgically resected specimens from patients with colon cancer, as previously described [1,19,20,21].

The anti-CEACAM6 mAb 9A6 was purchased from Thermo Fisher Scientific (Waltham, MA, USA).

### 2.3. Cell Lines and Culture 

The following human carcinoma cell lines were obtained from the American Type Culture Collection (ATCC) (Manassas, VA, USA): pancreatic cancer (CFPAC-1), acute myeloid leukemia (AML) (HL-60 and U937), chronic myeloid leukemia (CML) (K562) and human kidney embryo (HEK293T). All human carcinoma cell lines were maintained in a culture medium (Corning Life Science, Manassas, VA, USA) designated by the provider for propagation and maintenance. The culture medium was supplemented with 10% USA-sourced and heat-inactivated HyClone fetal bovine serum, defined (GE Healthcare Life Sciences, Issaquah, WA, USA), 100 U/mL penicillin and 100 μg/mL streptomycin (Corning Life Science, Manassas, VA, USA).

### 2.4. Isolation of Neutrophils from PBMCs 

The negative selection of neutrophils from PBMCs from healthy donors was performed using the EasySep™ Direct Human Neutrophil Isolation Kit (StemCell Technologies, Vancouver, BC, Canada), according to the manufacturer’s protocol. Neutrophils isolated from healthy donors were used for O-glycan profiling analysis and for the ADCC assay.

### 2.5. ELISA

To detect NEO-201 binding to the commercially available recombinant human (rh)CEACAM6 expressed in bacterial (*E. coli* BL21) (Origene, Rockville, MD, USA) and mammalian HEK293T cells (Acro Biosystem, Newark, DE, USA), 96-well ELISA plates were first coated overnight at 4 °C with 100 µL/well of 400 ng/mL recombinant human CEACAM6 (expressed in *E. coli* BL21 or in HEK293T cells) dissolved in 0.2 M carbonate-bicarbonate buffer pH 9.4. The plates were washed with 1× Tris-buffered saline (TBS) + 0.05% Tween-20 (VWR International, Radnor, PA, USA) (wash buffer) and then blocked with 200 µL/well of 5% milk in 1× TBS for 1 h at 37 °C. The plates were washed three times with wash buffer, and then 100 µL/well of NEO-201 or 9A6 mAb was added in two-fold serial dilution from 20 ng/mL to 0.016 ng/mL and incubated for 1 h at 37 °C. 

The plates were washed three times with wash buffer, and 100 µL/well donkey anti-human IgG antibody peroxidase labeled (VWR International, Radnor, PA, USA) was added to the plate at a 1:10,000 dilution and incubated for 1 h at 37 °C. The plates were washed three times with wash buffer, and 100 µL/well of tetramethylbenzidine (TMB) substrate solution (VWR International, Radnor, PA, USA) was added for 10 min at room temperature in the dark. The reaction was stopped by adding 50 µL/well of 2N H_2_SO_4_. Absorbance was measured at 450 nm on a FLUOstar Omega plate reader (BMG Labtech, Ortenmberg, Germany).

The amino acid sequences of the full-length CEACAM5 and CEACAM6 are shown in Figure S1.

### 2.6. Flow Cytometry

Cells to be profiled for the expression of O-glycans were selected for their reactivity with NEO-201 in flow cytometry. CFPAC-1, HL-60, U937 and K562 cell lines and human neutrophils were chosen as NEO-201 target cells. 

The cells were first incubated with 1 μL per test of LIVE/DEAD Fixable Aqua (Thermo Fisher Scientific, Waltham, MA, USA) in 1 mL of 1x phosphate buffered saline (PBS) (VWR International, Radnor, PA, USA) for 30 min at 4 °C to accomplish live-versus-dead-cell discrimination. Then, the cells were washed with 1x PBS and incubated with 2–5 μL of Human TruStain FcX™ (BioLegend, San Diego, CA, USA) in 100 uL of 1× PBS at room temperature for 5–10 min. To evaluate the reactivity of the cells with NEO-201, the cells were then stained in 100 μL of 1× PBS + 1% BSA (Teknova, Hollister, CA, USA) for 30 min at 4 °C with Pacific Blue conjugated NEO-201 (BioLegend, San Diego, CA, USA). 

After staining, the cells were washed twice with cold 1× PBS and examined using an FACSVerse flow cytometer (BD Biosciences, San Jose, CA, USA). The analysis of cellular fluorescence was performed using BD FACSuite software (BD Biosciences, San Jose, CA, USA). Positivity was determined by comparing unstained cells with cells stained with NEO-201. Staining values > 10% were considered positive.

### 2.7. O-Glycan Array to Identify O-Glycan Recognized by NEO-201 

An O-glycan array consisting of 94 O-glycans (Figure S2) was used to identify the O-glycans that bind NEO-201. The array layout is shown in Figure S3. The details of this procedure are described in the Supplementary Materials. The O-glycan array was obtained from Creative Proteomics (Shirley, NY, USA). NEO-201 was used at three concentrations (100 µg/mL, 20 µg/mL, 4 µg/mL) to incubate with the O-glycan array. The binding result was read using an Innopsys InnoScan 710 Microarray Scanner with a high-power laser at 5 PMT. Software was used to detect each spot on the array and calculate the relative fluorescence unit (RFU) intensity for each spot.

Background RFU was subtracted from each spot’s RFU value.

### 2.8. O-Glycan Profiling of Cells Reactive with NEO-201

NEO-201-reactive cell lines CFPAC-1, HL-60, U937, human neutrophils and NEO-201 non-reactive cell line K562 were used for O-glycan profile analysis. O-glycan profile analysis was performed by Creative Proteomics (Shirley, NY, USA). Several steps were used in the profiling procedure, including N-glycans removal, O-glycans preparation, permethylation and MS MALDI analysis [22]. The detail of the O-glycan profiling is described in the Supplementary Materials. 

### 2.9. Antibody-Dependent Cellular Cytotoxicity (ADCC) Assay

To evaluate the ADCC activity mediated by NEO-201 against HL-60 and human neutrophils, a modification of a previously described radioactive indium (In-111) release ADCC assay was performed [2]. 

To determine the NEO-201-mediated ADCC activity against HL-60, NK cells were isolated by negative selection from the PBMCs of two healthy donors using the EasySep human NK cell isolation kit (StemCell Technologies, Vancouver, BC, Canada), according to the manufacturer’s protocol. Purified NK cells were cultured overnight in RPMI 1640 medium (Corning Life Science, Manassas, VA, USA) supplemented with 10% USA-sourced and heat-inactivated HyClone fetal bovine serum, defined (GE Healthcare Life Sciences, Issaquah, WA, USA), 100 U/mL penicillin and 100 μg/mL streptomycin (Corning Life Science, Manassas, VA, USA) prior to being used as effector cells. On the day of the assay, target cells (1 × 10^6^) were labeled with 60 µCi of In-111. NK cells from two different healthy donors were added to the labeled target cells in the presence of 10 µg/mL of NEO-201 or 10 µg/mL of human IgG1 isotype (as control) at effector:target ratios 20:1 and 10:1 and incubated at 37 °C for 4 h. 

To determine the NEO-201-mediated ADCC activity against neutrophils, neutrophils were isolated from the whole blood of two healthy donors using the EasySep™ Direct Human Neutrophil Isolation Kit (StemCell Technologies, Vancouver, BC, Canada) and used as target cells. Isolated NK effectors from the same healthy donor were thawed the evening prior to conducting the assay and cultured overnight. On the day of the assay, neutrophils (1 × 10^6^) were used as the target and labeled with 60 µCi of In-111. Then, neutrophils were incubated at 37 °C for 4 h with 10 μg/mL of NEO-201 or human IgG1 isotype control antibody plus NK as effector cells at effector:target ratios 25:1, 12.5:1 and 6.25:1. 

Specific lysis was calculated as % specific lysis = 100 − [{average live target cell count for antibody-treated samples/average live target count for control samples} × 100]. 

### 2.10. Statistical Analysis

Data were analyzed using GraphPad Prism 8.0 (GraphPad Software, La Jolla, CA, USA). Student’s *t*-test, one-way ANOVA and two-way ANOVA with Bonferroni post-test analysis were performed where indicated, and *p* < 0.05 was considered statistically significant. 

## 3. Results

### 3.1. NEO-201 Binds to Mammalian-Expressed rhCEACAM6 but Not to Bacterial-Expressed rhCEACAM6 by ELISA 

In previous studies, we showed that NEO-201 specifically recognizes a tumor-associated variant of CEACAM5 and CEACAM6, which is not expressed in normal tissues, and proved that NEO-201 binds to both mammalian-expressed recombinant human CEACAM5 and CEACAM6 but not to CEACAM1 or CEACAM8 by ELISA [2,23]. 

To evaluate if post-translational modifications made on proteins by mammalian cells are responsible for the NEO-201 binding, we performed an ELISA comparing the binding of NEO-201 and the commercial anti-CEACAM6 mAb 9A6 to HEK293- expressed rhCEACAM6 (mammalian) or *E. coli*-expressed rhCEACAM6 (bacterial). 

**Figure 1:**
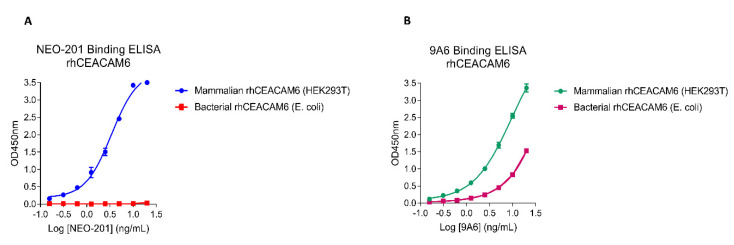
NEO-201 binding to mammalian rhCEACAM6 by ELISA. HEK293T: mammalian rhCEACAM6 (post-translational modification-competent). *E. coli*: bacterial rhCEACAM6 (post-translational modification-incompetent). rhCEACAM6 was used at 400 ng/mL. NEO-201 (**A**) and 9A6 (**B**) were used at two-fold dilutions down from 20 ng/mL.

Figure 1A shows that NEO-201 binds to mammalian-expressed rhCEACAM6 but not to bacterial-expressed rhCEACAM6, while 9A6 mAb binds to both types of rhCEACAM6 (Figure 1B)

These results further suggest that NEO-201 binds to a post-translational modification made by mammalian cells on rhCEACAM6.

### 3.2. NEO-201 Binds to O-Glycans 

Since glycosylation is a post-translational modification of proteins affected by oncogenesis and human cancer cells may have several glycan modifications, including the expression of truncated O-glycans, we performed an O-glycan array to test the binding activity of NEO-201 to 94 different O-glycan structures (Figure S2).

**Figure 2:**
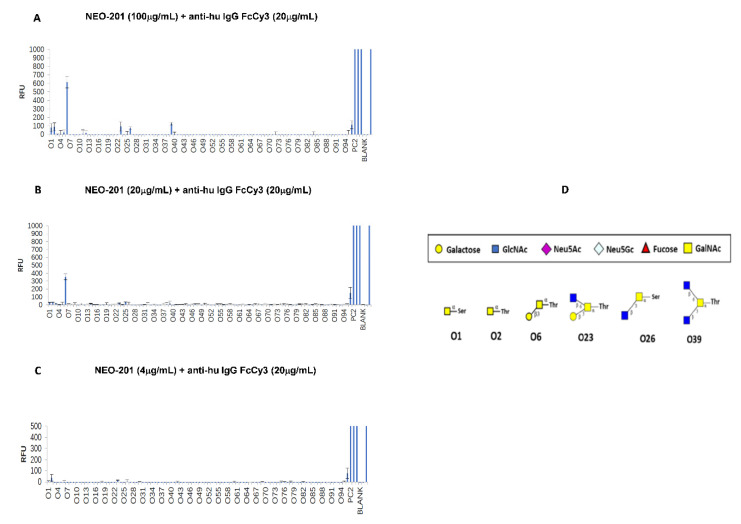
Analysis of NEO-201 binding to the O-glycan array. (**A**–**C**): NEO-201 was used at concentrations of 100, 20 and 4 µg/mL, and anti-human IgG FcCy3 was used at 20 µg/mL. The array was read using an Innopsys InnoScan 710 Microarray Scanner with a high-power laser at 5PMT. Software was used to detect each spot on the array and to calculate the relative fluorescence units (RFU) intensity for each spot. Background RFU was subtracted from each spot’s RFU value. The RFU scale is presented in vertical axes. (**D**): O-glycans recognized by NEO-201.

As shown in Figure 2, of these 94 O-glycans tested, 01, 02, 06, 023, 026 and 039 O-glycans showed binding to NEO-201 in a dose-dependent manner. The 06 binding interaction was the strongest of any observed. 01 and 02 are Tn antigens. 06 is core 1, 023 is core 2, 026 is core 3 and 039 is core 4 (Figure 2D). 

### 3.3. Human Cancer Cell Lines and Human Neutrophils Recognized by NEO-201 Express Core 1 and Extended Core 1 O-Glycans 

**Figure 3:**
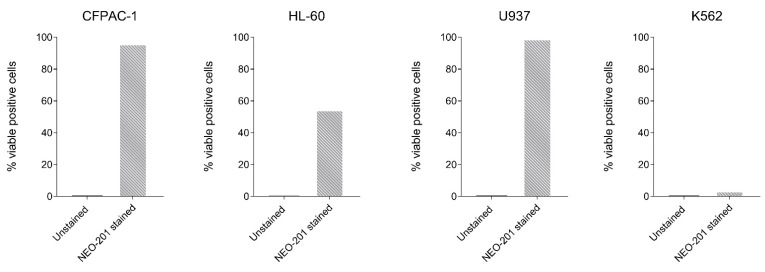
Flow cytometry analysis of the binding of NEO-201 to different cancer cell lines. Graphs depict the percentage of NEO-201 viable positive stained cells compared to the control unstained cells. NEO-201 positivity was defined as % positive cells >10%.

To further confirm that O-glycans are the real target of NEO-201, we profiled different human carcinoma cell lines for NEO-201 binding using flow cytometry. The assessment of the binding activity of NEO-201 revealed that CFPAC-1, HL-60 and U937 cancer cell lines show a strong reactivity for NEO-201, while K562 is negative (Figure 3). 

These cell lines were then screened to evaluate if they express O-glycans recognized by NEO-201. We also included neutrophils for the O-glycan profiling, since we observed in previous studies that they are targeted by NEO-201 [1,4]

O-glycan profiles for these cells, including *m*/*z*, compositions, proposed structures and the relative abundance of the most expressed O-glycans are reported in Table 1 and Table 2. The MS spectrum of the O-glycans of all cells screened is reported in Figure S4.

The O-glycan profile of the human pancreatic cancer cell line CFPAC-1 shows that this cell line expresses mostly sialylated core 1 O-glycans (Table 1).

The O-glycan profile of human neutrophils showed that these cells have a significant number of O-glycans in common with CFPAC-1, but the relative abundance varies (Table 1).

The O-glycan profile of the AML cell lines HL-60 and U937 and the CML cell line K562 showed that HL-60 only expresses the extended core 1 profile, U937 mostly expresses the extended core 1 and core 2 profiles and K562 only expresses the extended core 2 profile (Table 2).

The K562 cell line only expresses the extended core 2 glycans and is negative for the binding with NEO-201, while all the other cells positive for NEO-201 in flow cytometry mostly express core 1 and/or extended core 1 O-glycans. These data suggest that core 1 or extended core 1 O-glycans are epitopes recognized by NEO-201. 

### 3.4. NEO-201 Mediates ADCC to Kill Target Cells Expressing Core 1 and Extended Core 1 O-Glycans

In previous studies, we reported that NEO-201 can kill CFPAC-1 and other cancer cells through ADCC [1,2,3].

**Figure 4:**
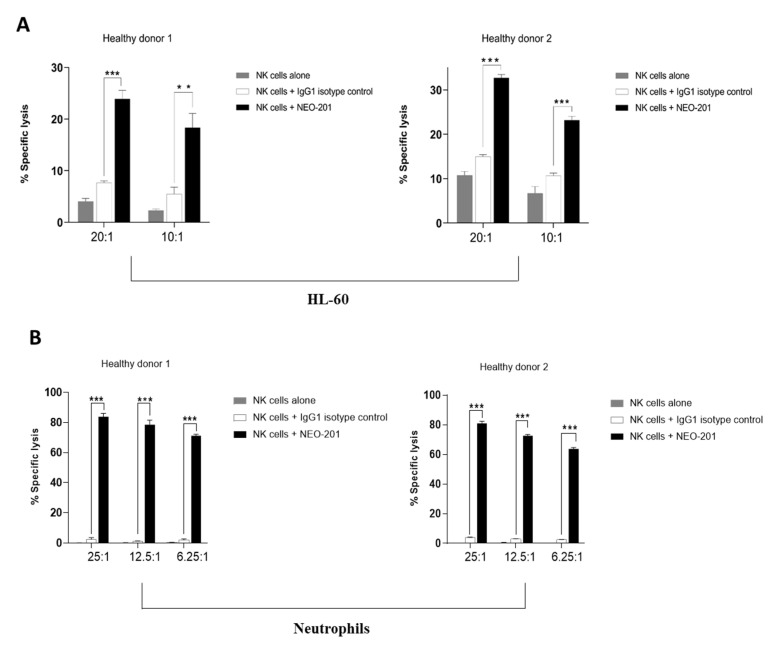
NEO-201 kills cancer cells expressing core 1 and/or extended core 1 O-glycans through ADCC. The HL-60 cell line and human neutrophils have been used as target cells to evaluate the ADCC mediated by NEO-201 in the ADCC assay. Cells were incubated with 10 µg/mL of NEO-201 or human IgG1 (negative control). NK cells from two healthy donors were used as effector cells at the indicated E:T ratios. Results are presented as the mean of % specific lysis ± SD (standard deviation) from three replicate wells in each experiment. *** statistically significant (*p* < 0.001) by two-way ANOVA (NEO-201 + NK cells vs. IgG1+ NK cells). ** statistically significant (*p* < 0.01) by two-way ANOVA (NEO-201 + NK cells vs. IgG1+ NK cells).

In this study, to further prove the concept that NEO-201 can kill target cells expressing core 1 and/or extended core 1 O-glycans through ADCC, we used HL-60 and human neutrophils as target cells in an in vitro ADCC assay. Human isolated NK cells from two different healthy donors were used as effector cells. The reason for not using U937 and K562 as a target is that both U937 and K562 are highly susceptible to NK cells killing and are thus not suitable to be used as a target for the ADCC assay. Figure 4 shows that NEO-201 can mediate ADCC activity against HL-60 and human neutrophils with isolated NK cells as effectors at all E:T ratios.

These data suggest that NEO-201 is able to target and kill cancer cells expressing core 1 and/or extended core 1 O-glycans through ADCC. 

## 4. Discussion

Glycosylation is an important post-translational modification made on mammalian proteins and lipids. Glycosylation is essential in various cellular processes such as cell–matrix interaction, cell-to-cell recognition and intracellular signaling, and glycosylation status is strongly affected by oncogenesis [24].

One glycosylation pattern disrupted in cancer cells is the O-glycosylation. The expression of incomplete/truncated O-glycans in cancer cells, especially those of epithelial origin such as breast, ovarian, gastric, pancreatic and colon cancers, is correlated with poor prognosis and tumor progression [14,15,16,17,18].

The aberrant expression of truncated O-glycans at the level of cancer cell proteins occurs when different saccharide components are added to the N-acetyl galactosamine (O-GalNAc) attached to the amino acids serine and threonine. In addition, the sialylation of the sugar of the glycan chain introduces additional diversity in the O-glycan repertoire expressed by cancer cells. All these aberrant O-glycans may serve as potential targets to improve the diagnosis and treatment of tumors and provide the molecular probes for their specific recognition [17,24,25,26,27]. The appearance of truncated O-glycans on the cancer cell surface is widely accepted as one of the characteristics of cancer [28]. Understanding how the immune system interacts with tumor-associated O-glycans will help in developing novel effective cancer immunotherapies. 

The hyper sialylation of O-glycans made by tumor cells could be one of the mechanisms used by tumors to escape from immune surveillance [29]. 

One promising strategy to subvert this mechanism and improve cancer immunotherapy efficacy is the employment of monoclonal antibodies that specifically recognize truncated O-glycans expressed in cancer cells. 

Several antibodies have been developed against O-glycans to counteract tumor growth. For example, in vivo experiments showed that mAbs against Tn-antigen use different mechanisms to kill tumor cells, including ADCC, CDC and the direct blocking of receptor signaling [30]. Examples of other anti-glycan antibodies used in pre-clinical studies and in clinical trials are the anti-GD3 ganglioside mAb R24 used to treat melanoma [31], antibodies against M2, fucosyl GM1, globo H and polysialic acid used to treat small cell lung cancers [32], the humanized mAb Hu3s193 for the treatment of patients with advanced epithelial cancers expressing the Lewis-Y antigen [33] and human monoclonal antibodies against the sialyl-Lewis (CA19.9) antigen for the treatment of different types of tumors [34]. Antibodies against CA19.9 can also be used for the detection and monitoring of advanced pancreatic cancer [35,36].

The first monoclonal antibody approved by the U.S. Food and Drug Administration (FDA) for the treatment of high-risk neuroblastoma was dinutuximab in 2015, a chimeric mAb recognizing the glycolipid GD2, a tumor-associated carbohydrate antigen (TACA) [37]. Furthermore, the National Cancer Institute has developed the Database of Anti-Glycan Reagents, where researchers reported the activity of more than 1100 anti-glycan antibodies [38]. In the context of anti-glycan mAbs, NEO-201 can represent a novel promising approach to treating cancer cells expressing O-glycans. 

In previous studies, we demonstrated that NEO-201 reacts to colon, ovarian, pancreatic, non-small cell lung, head and neck, cervical, uterine and breast cancers expressing tumor-associated variants of CEACAM5 and CEACAM6 but does not react to normal tissues [1,2,23]. NEO-201 can also bind to human regulatory T cells as well as human neutrophils, AML and MM cell lines in vitro [1,2,3,4,5,6]. 

NEO-201 has different mechanisms of action to kill target cancer cells, including ADCC, CDC and the blockade of the CEACAM5/CEACAM1 immune checkpoint inhibitory pathway [1,2,3,4]. 

In vivo, we demonstrated that NEO-201 attenuates the growth of human tumor xenografts in pancreatic and ovarian cancer murine models and shows safety/tolerability in non-human primates, with a transient decrease in neutrophils being the only adverse effect observed [1,2].

In addition, in a NEO-201 pharmacokinetics study performed in purpose-bred cynomolgus monkeys, we injected NEO-201 at dose levels of 5 mg/kg, 20 mg/kg and 49 mg/kg, and we observed quantifiable and dose-dependent serum concentrations of NEO-201 until 14 days post-dose. The mean half-life (HL) was 167 (20 mg/kg) or 170 (49 mg/kg) hours at higher doses [1]. Furthermore, NEO-201 was manufactured for clinical use (GMP), and stability tests performed on the NEO-201 drug product showed that NEO-201 has a long-term stability of 48 months. 

A first-in-human clinical trial using NEO-201 for the treatment of patients with metastatic colon cancer, pancreatic cancer, breast cancer, non-small cell lung cancer (NSCLC) and mucinous ovarian cancer, who were no longer eligible for standard therapy, has completed accrual and is currently undergoing data analysis [23]. 

Since NEO-201 binds to tumor-associated variants of CEACAM5 and CEACAM6 and CEACAM proteins are highly glycosylated proteins involved in cell migration, metastasis and drug resistance [7], in this study, we evaluated if NEO-201 can recognize specific glycan patterns attached on the protein structure of CEACAM5 and CEACAM6 during post-translational modifications made by mammalian cells. In this regard, we compared the binding of NEO-201 and of a commercially available anti-CEACAM6 mAb 9A6 to both mammalian and bacterial rhCEACAM6. As shown in Figure 1, NEO-201 binds to HEK293T cells (post-translational modification-competent mammalian cells) but not to rhCEACAM6 expressed by *E. coli* (post-translational modification-incompetent bacterial cells), while the 9A6 mAb binds to both types of rhCEACAM6. 

As described above, truncated O-glycans are highly expressed in cancer cells. Since NEO-201 binds only to mammalian rhCEACAM6 containing glycan patterns attached on the protein structure, we then evaluated the capacity of NEO-201 to recognize O-glycans expressed by cancer cells using different O-glycan arrays. 

Several methods have been used to evaluate antibodies specificity for glycans, such as ELISA, surface plasmon resonance (SPR) and glycan microarrays. Of these methods, glycan microarrays offer the highest throughput since they are composed of numerous glycan determinants or fragments of determinants, immobilized in a spatially defined arrangement [39]. 

In this study, using an O-glycan array consisting of 94 O-glycans, we demonstrated that NEO-201 recognizes six different O-glycans and that the strongest interaction is with a core 1 O-glycan (06) (Figure 2). These data were confirmed in the O-glycan array performed on cancer cell lines and human neutrophils positive for NEO-201 in flow cytometry. As shown in Table 1 and Table 2, cells positive for NEO-201 by flow cytometry express a high percentage of core 1 and/or extended core 1 O-glycans, while the CML cell line K562 (not recognized by NEO-201 in flow cytometry) expresses only core 2 O-glycans, suggesting that core 1 or extended core 1 O-glycans are epitopes recognized by NEO-201. We also demonstrated that cells expressing core 1 and/or extended core 1 O-glycans are killed by NEO-201 through ADCC. 

Our data confirm observations made in other studies. For example, Babu and colleagues reported that the human neutrophil glycome profile of the O-glycans consists of both core 1 and core 2 O-glycans with sialyl Lewis^x^ and Lewis^x^ as terminal epitopes [40]. In addition, Blöchl et al. showed that HL-60 and U937 AML cell lines predominantly express core 1 O-glycans [41]. Furthermore, both HL-60 and U937 do not express CEACAM5 and CEACAM6, suggesting that NEO-201 can bind to core 1 and extended core 1 O-glycans attached to any tumor-associated protein containing the amino acids serine and threonine.

Despite the available information regarding antiglycan antibodies, the identification and characterization of antiglycan antibodies remains a major challenge. 

One of the main limitations of antibodies targeting glycans is their low affinity for their target, which, in turn, reduces their antitumor efficacy. 

The enhancement of the antibody affinity for glycans is determined by conformational changes induced by the glycan in the peptide backbone. This phenomenon could partially affect the binding affinity of antiglycan antibodies targeting mixed epitopes composed of both glycan and the carrier molecule to which the glycan is attached, such as the peptide or lipid portions of the glycoproteins or glycolipids. An example of this phenomenon has been provided by Martínez-Sáez et al., who demonstrated that the binding of the antibody SM3 to glycopeptides bearing the α-O-GalNAc-Thr and α-O-GalNAc-Ser antigens is affected by distinct atomic presentations of the Tn-carrying serine (α-GalNAc-Ser) and threonine (α-GalNAc-Thr) antigens when bound to SM3 [42]. Furthermore, it has been demonstrated that antiglycan antibody binding between a single binding site and a single glycan determinant is usually weak, with equilibrium dissociation constants (KD) in the micromolar to low millimolar range. The multivalent binding of antibodies engaging two or more binding sites with two or more glycan determinants can achieve a much higher functional affinity [43,44,45]. 

The KD of NEO-201 is 5.49 × 10^−9^ with Rmax = 99.3 and Chi2 = 3.47 (unpublished data), allowing NEO-201 to have a strong binding affinity for its target. In this regard, here, we have demonstrated that NEO-201 binds to core 1 and/or core 1 extended O-glycans expressed in its target cells. O-glycans can be attached to amino acid regions containing serine and threonine on proteins. This study suggests that NEO-201 could be a good anti-glycan mAb for the treatment of different types of cancers not only expressing core 1 and/or extended core 1 O-glycans attached to the tumor-associated variant of CEACAM5 and CEACAM6 but also to other tumor-associated proteins. Further studies will evaluate if core-1 and/or extended core-1 O-glycans recognized by NEO-201 are attached to other specific tumor-associated proteins (beyond CEACAM5 and CEACAM6) expressed by the different types of tumors targeted by NEO-201. 

## 5. Conclusions

This study demonstrated that NEO-201 recognizes different types of O-glycans, with the strongest binding to core 1 and extended core 1 O-glycans. NEO-201-reactive cells such as the pancreatic cell line CFPAC-1, human neutrophils and the human hematological neoplastic cells HL60 and U937 express mostly core 1 and/or extended core 1 O-glycan profiles. These cells can be killed by NEO-201 through NK-mediated ADCC.

The aberrant expression of O-glycans is associated with cancer development, progression, invasion and metastasis in both solid tumors of epithelial origin [14,15,16,17,18] and hematological malignancies such as AML and multiple myeloma [46,47]. The employment of NEO-201, which has a strong binding affinity for tumor-associated proteins carrying core 1 and/or extended core 1 O-glycans, could represent a novel and promising tool in the immunotherapy treating both solid and liquid tumors. 

## Figures and Tables

**Table 1:** O-glycan profile and relative abundance of CFPAC-1 and human neutrophils.

m/z Observed		Proposed Structure	Relative Abundance (%)	O-Glycan Profile
Theoretical	Observed			
CFPAC-1 (human pancreatic cell line)				
895.5	895.5	* 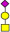 *	74.38%	Sialyl-T antigen (Sialyl core 1)
534.3	534.3	* 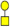 *	13.92%	T antigen (core 1)
1256.7	1256.7	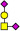	7.86%	Disialyl-T antigen (Disialyl core 1)
Human neutrophils				
895.5	895.5	* 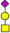 *	33.08%	Sialyl-T antigen (Sialyl core 1)
534.3	534.3	* 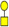 *	24.65%	T antigen (core 1)
1256.7	1256.7	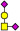	11.81%	Disialyl-T antigen (Disialyl core 1)
1705.9	1705.9	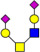	6.25%	Extended core 2
983.6	983.6	* 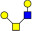 *	5.03%	
1344.8	1344.8	* 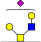 *	4.02%	
779.4	779.4	* 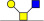 *	2.73%	Core 2
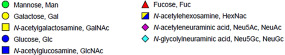				

Data are presented as the percentage of the most representative O-glycans. The legend below the table depicts monosaccharides contained in each O-glycan structure reported in the table.

**Table 2:** O-glycan profile and relative abundance of HL-60, U937 and K562 cell lines.

m/z Observed		Proposed Structure	Relative Abundance (%)	O-Glycan Profile
Theoretical	Observed			
HL-60 (human acute myeloid leukemia cell line)				
1758.8	1758.8	* 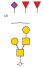 *	100%	Extended core 1
U937 (human acute myeloid leukemia cell line)				
1705.9	1705.9	* 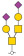 *	73.05%	Extended core 1
1879.9	1879.9	* 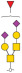 *	12.10%	
1821.9	1821.9	* 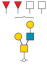 *	8.23%	Extended core 2
1722.9	1722.9	* 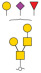 *	6.62%	Extended core 1
K562 (human chronic myeloid leukemia cell line)				
1821.9	1821.9	* 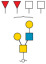 *	100%	Extended core 2
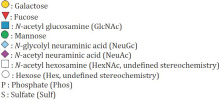				

Data are presented as the percentage of the most representative O-glycans. The legend below the table depicts monosaccharides contained in each O-glycan structure reported in the table.

## Data Availability

The data are contained within the article.

## Supplementary Materials

The following supporting information can be downloaded at: https://www.mdpi.com/article/10.3390/cancers14204999/s1, Figure S1: Amino acid sequences of the full length CEACAM5 and CEACAM6; Figure S2: O-glycan structure of the 94 different O-glycans used in the O-glycan array to test the binding activity of NEO-201 to O-glycans; Figure S3: O-glycan array layout used to test the binding activity of NEO-201 to O-glycans; Figure S4: MS spectrum of O-glycans detected on all cells screened to elucidate O-glycan profiles of NEO-201 reactive cells.
