# Supplementary material for: Identification of the O-Glycan Epitope Targeted by the Anti-Human Carcinoma Monoclonal Antibody (mAb) NEO-201
Source: Cancers (Basel). 2022 Oct 12;14(20):4999. doi: 10.3390/cancers14204999 (PMC9599200; doi:10.3390/cancers14204999)
Supplement: Supplementary file 1 [file cancers-14-04999-s001.zip › supplemental figures.pdf]

CEACAM5 (NM_004363.5)

```
        10         20         30         40         50
MESPSAPPHR WCIPWQRLLL TASLLTFWNP PTTAKLTIES TPFNVAEGKE
        60         70         80         90        100
VLLLVHNLPQ HLFGYSWYKG ERVDGNRQII GYVIGTQQAT PGPAYSGREI
       110        120        130        140        150
IYPNASLLIQ NIIQNDTGFY TLHVIKSDLV NEEATGQFRV YPELPKPSIS
       160        170        180        190        200
SNNSKPVEDK DAVAFTCEPE TQDATYLWWV NNQSLPVSPR LQLSNGNRTL
       210        220        230        240        250
TLFNVTRNDT ASYKCETQNP VSARRSDSVI LNVLYGPDAP TISPLNTSYR
       260        270        280        290        300
SGENLNLSCH AASNPPAQYS WFVNGTFQQS TQELFIPNIT VNNSGSYTCQ
       310        320        330        340        350
AHNSDTGLNR TTVTTITVYA EPPKPFITSN NSNPVEDEDA VALTCEPEIQ
       360        370        380        390        400
NTTYLWWVNN QSLPVSPRLQ LSNDNRTLTL LSVTRNDVGP YECGIQNELS
       410        420        430        440        450
VDHSDPVILN VLYGPDDPTI SPSYTYYRPG VNLSLSCHAA SNPPAQYSWL
       460        470        480        490        500
IDGNIQQHTQ ELFISNITEK NSGLYTCQAN NSASGHSRTT VKTITVSAEL
       510        520        530        540        550
PKPSISSNNS KPVEDKDAVA FTCEPEAQNT TYLWWVNGQS LPVSPRLQLS
       560        570        580        590        600
NGNRTLTLFN VTRNDARAYV CGIQNSVSAN RSDPVTLDVL YGPDTPIISP
       610        620        630        640        650
PDSSYLSGAN LNLSCHSASN PSPQYSWRIN GIPQQHTQVL FIAKITPNNN
       660        670        680        690        700
GTYACFVSNL ATGRNNSIVK SITVSASGTS PGLSAGATVG IMIGVLVGVA

LI
```

CEACAM6 (NM_002483.6)

```
        10         20         30         40         50
MGPPSAPPCR LHVPWKEVLL TASLLTFWNP PTTAKLTIES TPFNVAEGKE
        60         70         80         90        100
VLLLAHNLPQ NRIGYSWYKG ERVDGNRLIV GYVIGTQQAT PGPAYSGRET
       110        120        130        140        150
IYPNASLLIQ NVTQNDTGFY TLQVIKSDLV NEEATGQFHV YPELPKPSIS
       160        170        180        190        200
SNNSNPVEDK DAVAFTCEPE VQNTTYLWWV NGQSLPVSPR LQLSNGNMTL
       210        220        230        240        250
TLLSVKRNDA GSYECEIQNP ASANRSDPVT LNVLYGPDVP TISPSKANYR
       260        270        280        290        300
PGENLNLSCH AASNPPAQYS WFINGTFQQS TQELFIPNIT VNNSGSYMCQ
       310        320        330        340
AHNSATGLNR TTVTMITVSG SAPVLSAVAT VGIMIGVLAR VALI
```

Figure S1. Amino acid sequences of the full length CEACAM5 and CEACAM6.

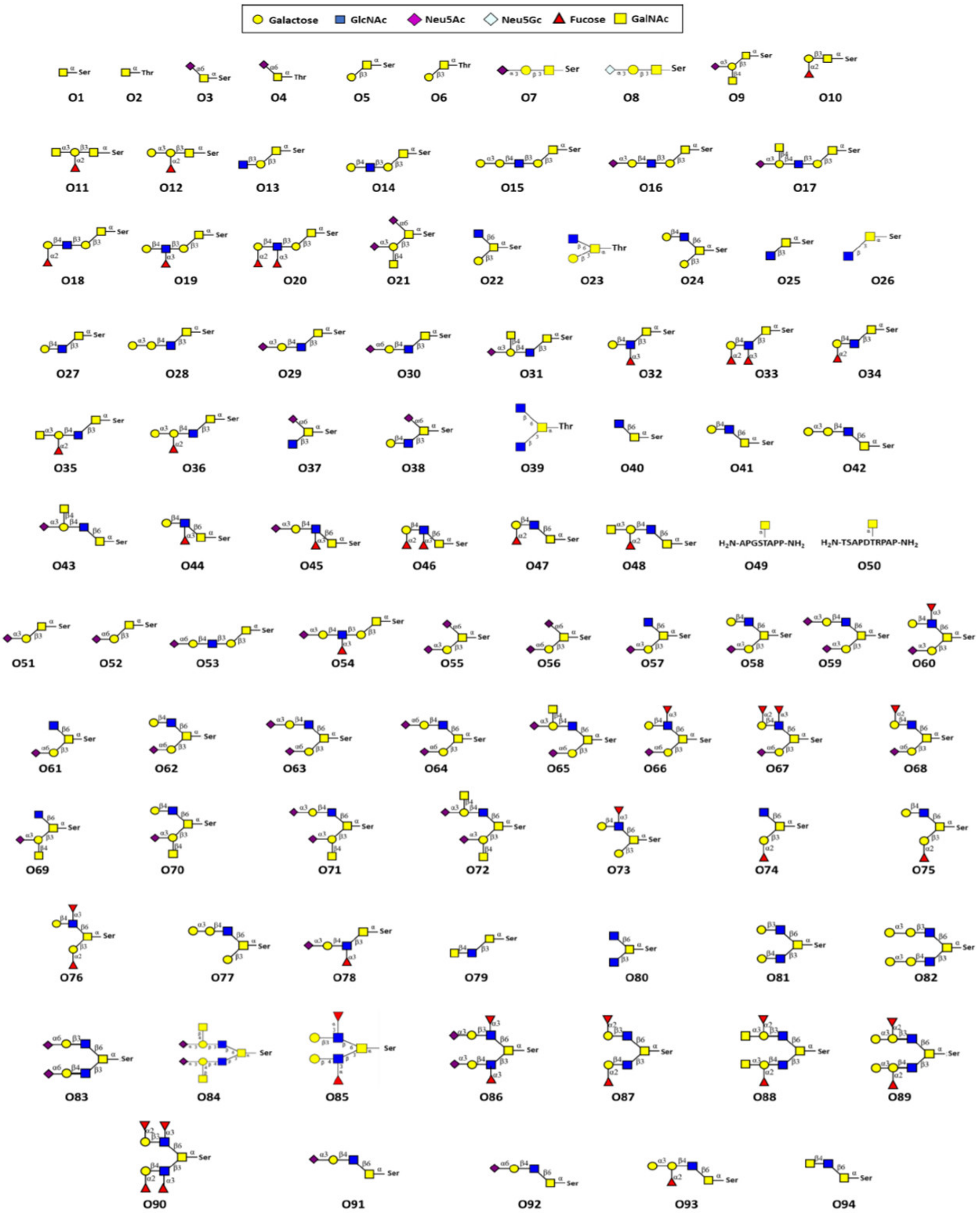

Figure S2. O-glycan structure of the 94 different O-glycans used in the O-glycan array to test the binding activity of NEO-201 to O-glycans.

1	1	1	2	2	2	3	3	3	4	4	4	5	5	5	6	6	6
7	7	7	8	8	8	9	9	9	10	10	10	11	11	11	12	12	12
13	13	13	14	14	14	15	15	15	16	16	16	17	17	17	18	18	18
19	19	19	20	20	20	21	21	21	22	22	22	23	23	23	24	24	24
25	25	25	26	26	26	27	27	27	28	28	28	29	29	29	30	30	30
31	31	31	32	32	32	33	33	33	34	34	34	35	35	35	36	36	36
37	37	37	38	38	38	39	39	39	40	40	40	41	41	41	42	42	42
43	43	43	44	44	44	45	45	45	46	46	46	47	47	47	48	48	48
49	49	49	50	50	50	51	51	51	52	52	52	53	53	53	54	54	54
55	55	55	56	56	56	57	57	57	58	58	58	59	59	59	60	60	60
61	61	61	62	62	62	63	63	63	64	64	64	65	65	65	66	66	66
67	67	67	68	68	68	69	69	69	70	70	70	71	71	71	72	72	72
73	73	73	74	74	74	75	75	75	76	76	76	77	77	77	78	78	78
79	79	79	80	80	80	81	81	81	82	82	82	83	83	83	84	84	84
85	85	85	86	86	86	87	87	87	88	88	88	89	89	89	90	90	90
91	91	91	92	92	92	93	93	93	94	94	94	NC	NC	NC	PC1	PC1	PC1
PC2	PC2	PC2	PC3	PC3	PC3	PC4	PC4	PC4	Blank	Blank	Blank	Blank	Blank	Blank	Marker	Marker	Marke

Figure S3. O-glycan array layout used to test the binding activity of NEO-201 to O-glycans.

PC1:B1-PEG-NH2; PC2: human IgG; PC3: mouse IgG; PC4: Rabbit IgG
NEO-201 was used at three concentrations (100µg/mL, 20µg/mL, 4µg/mL) and was incubated with O-glycans in the O-glycan arrays for 1 h at room temperature. The arrays were then washed and incubated with anti-human IgG FcCy3 at a concentration of 20µg/mL for 1 h at room temperature.
Arrays were read using an Innopsys InnoScan 710 Microarray Scanner with a high-power laser at 5PMT. Software was used to detect each spot on the array and calculate the relative fluorescence units (RFU) intensity for each spot. Background RFU was subtracted from each spot's RFU value.

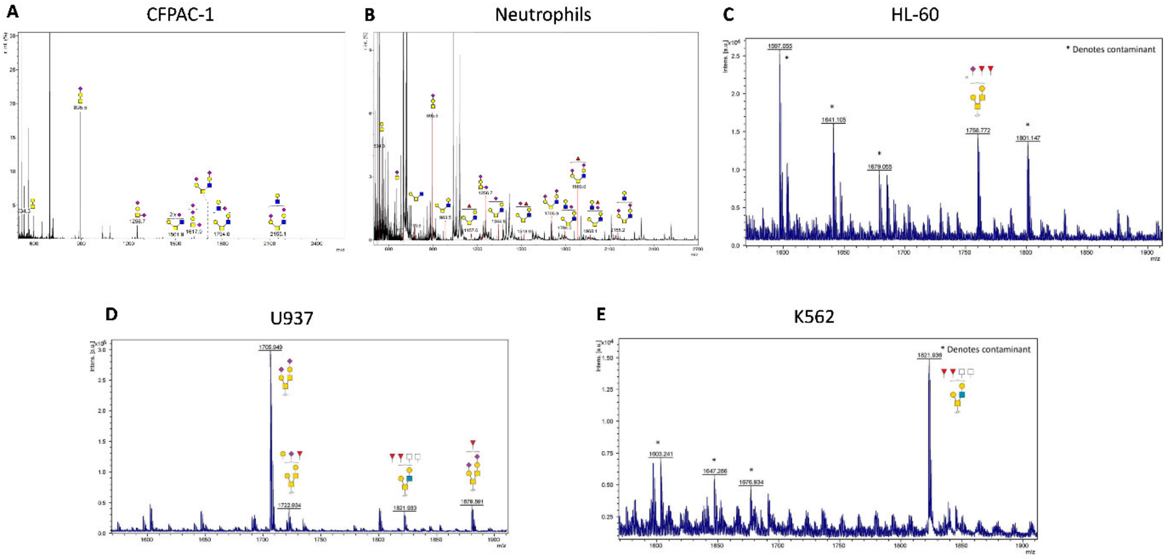

Figure S4. MS spectrum of O-glycans detected on all cells screened to elucidate O-glycan profiles of NEO-201 reactive cells.

A. pancreatic cancer cell line CFPAC-1. B. Human neutrophils. C. acute myeloid leukemia cell line HL-60. D. Acute myeloid leukemia cell line U937. E. Chronic myeloid leukemia cell line K562.

MS data was acquired on a Bruker UltraFlex II MAL DI-TOF Mass Spectrometer instrument. Reflective positive mode was used, and data were recorded between 500 m/z and 4000 m/z for O-glycans.

For each MS O-glycan profiles the aggregation of 20,000 laser shots or more were considered for data extraction. Mass signals of a signal/noise ratio of at least 2 were considered and only MS signals matching an O-glycan composition were considered for further analysis and annotated. Subsequent MS post-data acquisition analysis were made using mMass.
