# Supplementary material for: Identification of the O-Glycan Epitope Targeted by the Anti-Human Carcinoma Monoclonal Antibody (mAb) NEO-201
Source: Cancers (Basel). 2022 Oct 12;14(20):4999. doi: 10.3390/cancers14204999 (PMC9599200; doi:10.3390/cancers14204999)
Supplement: Supplementary file 1 [file cancers-14-04999-s001.zip › supplemental materials and methods.pdf]

Supplement 1: Identification of O-glycans binding to NEO-201 using O-glycan Arrays

An O-glycan array consisting of 94 O-glycans (Figure S2) was employed to identify the O-glycans that bind NEO-201. The array was blocked for 30 min using a Glycan Array Blocking Buffer (GAAB, TBS-T based buffer). Then the array was washed 3 times using the GAAB). The samples were diluted in GAAB to the desired concentrations, and then applied directly to the array. The array was covered and shaken at 80 rpm for 1 h at room temperature. The array was then washed 3 times again with GAAB, and then NEO-201 was diluted in GAAB and used at three concentrations (100μg/mL, 20μg/mL and 4μg/mL) to incubate with the O-glycan Array for 1 h at room temperature covered from light and shaken at 80 rpm. The Arrays were then washed and incubated with anti-human IgG FcCy3 at a concentration of 20μg/mL for 1 h at room temperature covered from light and shaken at 80 rpm. The Arrays were then washed 3x with GAAB and 2x with MilliQ water. The array was then read using an Innopsys InnoScan 710 Microarray Scanner with a high-power laser at 5PMT. Software was used to detect each spot on the array and calculate the relative fluorescence units (RFU) intensity for each spot. Background RFU was subtracted from each spot's RFU value. The median of each glycan's spot was determined and graphed.

Supplement 2: Evaluation of O-glycan profiles of NEO-201 reactive cells and estimation of the relative abundance of each O-glycan detected

N-glycans removal:

After 3 washes in PBS the cell pellet was resuspended in 1 mL of lysis buffer (25 mM TRIS, 150mM NaCl, 5mM EDTA, 0.5% w/v CHAPS, pH 7.4) and sonicated (5 pulses of 10 s). The lysed sample was next dialyzed against 50 mM ammonium bicarbonate for 24 h at 4 °C and with the dialysis buffer changed three times. After lyophilization, the dialyzed material was resuspended in 1 mL of a 2 mg/mL DTT (1,4-Dithiothreitol) solution and incubated at 50 °C for 90 min. Then 0.5 mL of 12 mg/mL solution of iodoacetamide in 0.6 M TRIS buffer pH 8.5 was added to the DTT-treated sample and sample were incubated at room temperature in the dark for 90 min.

Sample were then dialyzed against 50 mM ammonium bicarbonate at 4 °C for 16h-24h, changing the buffer 3 times. The molecular cut-off should be between 1 and 5 kDa. After dialysis samples were transferred into a 15 mL tubes and lyophilized.

Dry samples were then resuspended in 0.5 mL of a 50 µg/mL solution of TPCK-treated trypsin in 50 mM ammonium bicarbonate and incubated overnight (12-16 h) at 37 °C. Reaction was stopped by adding 2 drops of 5% acetic acid.

The trypsin-digested samples were then added onto a C18 Spe-Pak (50 mg) column with methanol, 5% acetic acid, 1-propanol and 5% acetic acid. Column was then washed with 4 ml of 5% acetic acid and peptides were eluted from the C18 column with 2 ml of 20% 1-propanol, then 2 ml 40% 1-propanol and finally 2 ml of 100% 1-propanol. All the eluted fractions were pooled, and samples were lyophilized.

Dried samples were then resuspended thoughtfully in 200 µL of 50 mM ammonium bicarbonate and 2 µL of PNGaseF and incubated at 37 °C for 4 h. Then, another 3 µL of PNGaseF was added for overnight (12-16 h) incubation at 37 °C. Reaction was stopped by adding 2 drops of 5% acetic acid.

The PNGaseF-digested sample were added onto a C18 Spe-Pak (50 mg) column with Methanol, 5% acetic acid, 1-Propanol and 5% acetic acid and the flow through was discarded.

O-glycans preparation:

The material bound to the column (containing peptides and potentially O-glycopeptides) was collected by sequential elutions with 1 mL of 20% 1-propanol, 1 mL of 40% 1-propanol and 1 mL of 100% 1-propanol. The eluted fractions were then lyophilized. 400 µL of a 55 mg/mL NaBH4 (sodium borohydride) in 0.1 M NaOH solution were then added to the samples and samples were incubated at 45 °C overnight.
The reaction was terminated by adding 3-4 drops of pure (100%) acetic acid until fizzing stops.
The acetic acid-neutralized samples were loaded onto a Dowex (50W X8, mesh size 200-400) ion exchange resin column previously washed with 10 ml of 5% acetic acid. After loading the column was then washed with 3 mL of 5% acetic acid. Flow through was then collected, and pooled. The collected material was lyophilized. 1 mL of acetic acid: methanol solution (1:9; v/v=10%) was then added to the lyophilized sample. Samples were then vortexed thoroughly and dried under a stream of nitrogen for three times.
The co-evaporated dried samples were then resuspended in 200 µL of 50% methanol and loaded onto a C18 Spe-Pak (50 mg) column conditioned with methanol, 5% acetic acid, 1-propanol and 5% acetic acid. After loading the column was washed with 4 mL of 5% acetic acid and flow through was collected and pooled. The collected material was lyophilized before to proceed to permethylation.

Permethylation:

The Preparation of the slurry NaOH/DMSO solution is made fresh every time. Mortar, pestle, and glass tubes were washed with Milli Q water and dried beforehand. Whenever possible, liquid reagents were handled with disposable glass pipettes. Solvents are HPLC grade or higher.
7 pellets of NaOH were grinded with a clean and dry mortar and pestle in 3 mL of DMSO. 1 mL of this slurry solution was added to dry sample in a glass tube with a screw cap. Then, 500 µL of Iodomethane were added, the lid was closed tightly, and the sample was shaked at room temperature for ~30 min. Then, the cap was open slowly as gas pressure has built up and 1 mL of MilliQ water was added to stop the reaction. Tube was vortexed until all solid is dissolved.
Then, 1 mL of Chloroform and additional 3 mL of MilliQ water were added, tubes were vortexed thoroughly to mix both phases and centrifuged briefly to separate the chloroform and the water phases (~5000 rpm, <20 sec). The aqueous top layer was discarded, and the wash was repeated 2 more times adding 3 ml of Milli Q water. The chloroform fraction was dried with a SpeedVac (~20-30 min). Then, the dried sample were resuspended with with 200 µL of 50% methanol and loaded onto a C18 Spe-Pak (200 mg) column conditioned with Methanol, Acetonitrile and MilliQ water. After loading, the column was washed with 2 mL of 15% acetonitrile and then the column was eluted in a clean glass tube with 3 mL of 50% acetonitrile. The eluted fractions were then lyophilized for MS analysis.

MS MALDI analysis:

MS data was acquired on a Bruker UltraFlex II MAL DI-TOF Mass Spectrometer instrument. Reflective positive mode was used, and data were recorded between 500 m/z and 4000 m/z for O-glycans.

For each MS O-glycan profiles the aggregation of 20,000 laser shots or more were considered for data extraction. Mass signals of a signal/noise ratio of at least 2 were considered and only MS signals matching an O-glycan composition were considered for further analysis and annotated. Subsequent MS post-data acquisition analysis were made using mMass.
